# 1,25-Dihydroxyvitamin D_3_ and Its Analog TX527 Promote a Stable Regulatory T Cell Phenotype in T Cells from Type 1 Diabetes Patients

**DOI:** 10.1371/journal.pone.0109194

**Published:** 2014-10-03

**Authors:** Tom L. Van Belle, An-Sofie Vanherwegen, Dorien Feyaerts, Pierre De Clercq, Annemieke Verstuyf, Hannelie Korf, Conny Gysemans, Chantal Mathieu

**Affiliations:** 1 Clinical and Experimental Endocrinology (CEE), KU Leuven, Leuven, Belgium; 2 Laboratory for Organic Synthesis, Department of Organic Chemistry, Ghent University, Ghent, Belgium; La Jolla Institute for Allergy and Immunology, United States of America

## Abstract

The emergence of regulatory T cells (Tregs) as central mediators of peripheral tolerance in the immune system has led to an important area of clinical investigation to target these cells for the treatment of autoimmune diseases such as type 1 diabetes. We have demonstrated earlier that *in vitro* treatment of T cells from healthy individuals with TX527, a low-calcemic analog of bioactive vitamin D, can promote a CD4^+^CD25^high^CD127^low^ regulatory profile and imprint a migratory signature specific for homing to sites of inflammation. Towards clinical application of vitamin D-induced Tregs in autologous adoptive immunotherapy for type 1 diabetes, we show here that 1,25-dihydroxyvitamin D_3_ [1,25(OH)_2_D_3_] and TX527 similarly imprint T cells from type 1 diabetes patients with a CD4^+^CD25^high^CD127^low^ regulatory profile, modulate surface expression of skin- and inflammation-homing receptors, and increase expression of CTLA-4 and OX-40. Also, 1,25(OH)_2_D_3_ and TX527 treatment inhibit the production of effector cytokines IFN-γ, IL-9, and IL-17. Importantly, 1,25(OH)_2_D_3_ and TX527 promote the induction of IL-10-producing CD4^+^CD25^high^CD127^low^ T cells with a stable phenotype and the functional capacity to suppress proliferation of autologous responder T cells *in vitro*. These findings warrant additional validation of vitamin D-induced Tregs in view of future autologous adoptive immunotherapy in type 1 diabetes.

## Introduction

The incidence of type 1 diabetes, in particular in children, is rising globally and rapidly, making it an important health challenge worldwide. Current treatment for the acute symptoms, such as hyperglycemia, is life-long insulin supplementation or replacement. Nevertheless, long-term complications such as kidney failure, blindness, heart disease and stroke still occur, emphasizing the need for novel therapies that can revert or halt disease progression. So far, none of the immune-based interventions have been reported to provide long-term disease remission without affecting normal immunity. Breakdown of the mechanisms that maintain immune tolerance allow autoreactive T cells to play a pivotal role in disease pathogenesis [1]–[3]. The emergence of regulatory T cells (Tregs) as essential “masters of immune regulation” [4], because they can potently and selectively counteract the progressive loss of immune tolerance [5], has led to the development of Treg-based cellular therapies for the treatment of autoimmune diseases, transplantation, and graft versus host disease [5].

In type 1 diabetes, conflicting data are reported on whether patients have lower [6] or normal [7], [8] frequencies of Tregs and on whether patients have Tregs with defective [9], [10] or normal [8] functional capacity. Yet, targeting the frequency and/or function of Tregs – whether it is directly *in vivo* or by *ex vivo* expansion followed by autologous adoptive immunotherapy – provides the advantage of restoring the balance in the immune system without a generalized immunosuppression. Preclinical studies indeed support this: for example, adoptive transfer of Tregs expanded *in vitro* can prevent and even reverse diabetes in non-obese diabetic (NOD) mice [11]. In addition, human Tregs can be isolated from newly-onset type 1 diabetes patients and expanded *in vitro* with anti-CD3 and anti-CD28 in the presence of high doses of recombinant IL-2 [12]. A phase 1 clinical trial currently tests the safety and efficacy of intravenous infusion into type 1 diabetes patients of autologous polyclonal Tregs expanded *in vitro* (NCT01210664). However, the inclusion of additional immunomodulatory agents during *ex vivo* expansion to limit any inflammatory potential of expanded Tregs may be warranted [13].

Vitamin D, in particular its active metabolite 1,25(OH)_2_D_3_, is an immunomodulator [14], [15] and a wide variety of immune cells express the nuclear vitamin D receptor (VDR) as well as vitamin D-activating enzymes [16], [17]. Most reports on 1,25(OH)_2_D_3_ underscore its actions on antigen presenting cells as the key feature underlying the immunomodulatory properties [18], [19], but activated T cells also express VDRs [20]. We and others have recently shown that 1,25(OH)_2_D_3_ and the low-calcemic analog TX527 can directly affect human T cells, inhibiting the production of proinflammatory cytokines, imprinting a migratory signature specific for homing to sites of inflammation and promoting a Treg profile and function [21], [22]. Clinical use of such vitamin D-induced Tregs relies on autologous adoptive immunotherapy and thus on successful immunomodulation of T cells from type 1 diabetes patients. In this study we found that indeed, exposure to 1,25(OH)_2_D_3_ or TX527 inhibits effector cytokine production and imprints a stable Treg profile on human T cells with suppressive capacity on autologous T cells, both from control donors and type 1 diabetes patients.

## Materials and Methods

### Donors and study design

Control individuals were recruited from the general population at KU Leuven (Leuven, Belgium). Patients with established type 1 diabetes, diagnosed on the basis of clinical criteria [23] and the presence of autoantibodies, were recruited from the clinical department of Endocrinology at the University Hospital Leuven. Duration of diabetes was 14.9±.6 years and glycated haemoglobin A1c (HbA1C) of 8.0±1.1%. Approximately 30 mL of blood was collected in EDTA-coated tubes (BD Biosciences, Erembodegem, Belgium). A sample of serum and cell pellet was stored at −80°C for biobanking purposes. One part of the peripheral blood mononuclear cells (PBMCs) was cryopreserved in freezing medium (50% RPMI, 40% FCS and 10% DMSO) using a cold freezing protocol. Ethical approval for this study was granted by the Ethics Committee of the KU Leuven (S52697) and written informed consent obtained.

### mAbs and reagents

T cell medium consisted of RPMI 1640 medium, supplemented with 10% heat-inactivated FCS, penicillin (100 IU/mL) and streptomycin (100 IU/mL) (Invitrogen, Ghent, Belgium). Purified anti-CD3 (UCHT1), anti-CD28 (37407) and human recombinant IL-1β were from R&D Systems (Minneapolis, USA). Human recombinant IL-2, human recombinant IL-6, and human recombinant TGF-β were from PeproTech (Rocky Hill, USA). TX527 [19-nor-14,20-bisepi-23-yne-1,25(OH)_2_D_3_], a 1,25(OH)_2_D_3_-analog, was synthesized by M. Vandewalle and P. De Clercq (University of Ghent, Ghent, Belgium) and obtained from Théramex S.A. (Monaco, France). 1,25(OH)_2_D_3_ (calcitriol) was from Sigma-Aldrich (St. Louis, USA).

### T cell isolation and culture

PBMCs were isolated by Percoll-gradient centrifugation (Axis-Shield Pc AS, Oslo, Norway) from fresh whole blood samples. CD3^+^ T cells were purified from PBMCs by negative selection using the Pan T cell Isolation Kit II (Miltenyi Biotec, Bergisch Gladbach, Germany) according to manufacturer's protocol (purity always>96%). Purified CD3^+^ T cells (1×10^6^ cells/mL) were cultured in 24-well culture plates in T cell medium with plate-bound anti-CD3 and anti-CD28 (both 1 µg/mL). Every other day, cells were split and supplemented with fresh T cell medium containing human rIL-2 (125 U/mL) and treated with 1,25(OH)_2_D_3_, TX527 (10^−8^ M) or vehicle (ethanol) starting at day 2. This treatment schedule is chosen based on previous results showing an optimal induction of VDR expression on day 2 following activation [22], [24]. To evaluate the stability of the induced Treg population, cells were stimulated with a cytokine cocktail containing 50 ng/ml rIL-1β, 125 U/ml rIL-2, 50 ng/ml rIL-6 and 3 ng/ml TGF-β during the last 48 h of the T cell culture as previously described [25], [26].

### Analysis of cell populations and isolation for functional studies

Tregs in PBMCs at baseline were quantified using flow cytometry after staining with anti-CD4-APC, anti-CD8-eFluor780 (eBioscience, San Diego, CA), anti-CD25-PE (Miltenyi) and anti-CD127-PECy7 (BD Biosciences). Staining for surface protein was done with anti-CD4-Pacific Orange (Life Technologies), anti-CD8-APC-eFluor780 (eBioscience), anti-CD45RO-PerCP-eFluor710 (eBioscience), anti-OX40-FITC (BD Biosciences), anti-CCR10-PE (R&DSystems), anti-CXCR6-AF647 (BioLegend), and anti-CLA-Pacific Blue (Life Technologies) (30 min, 37°C). For FOXP3/CTLA-4 intracellular staining, cells were incubated with anti-CD4-Pacific Orange, anti-CD8-APC-eFluor780, anti-CD25-PE, anti-CD127-PECy7, and anti-CTLA4-APC (BD Biosciences)(20 min, 4°C), then fixed and permeabilized using the human FOXP3 Buffer Set (BD Biosciences), according to manufacturer's instructions, and incubated with anti-FOXP3-AF488 (eBioscience) and anti-CTLA4-APC (30 min, RT). Samples were acquired on a Gallios flow cytometer (Beckman Coulter) and analyzed with FlowJo (TreeStar, Ashland, USA). For RNA preparation or functional studies, CD4^+^CD25^high^CD127^low^ cells were sort-purified from cultures using a FACSAria™ I cell sorter (BD Biosciences). Moreover, for the functional studies, CD4+ T cells were magnetically isolated from T cell cultures using the human CD4+ T cell isolation kit (Miltenyi) according to the manufacturer's guidelines.

### RNA isolation and real-time RT-PCR

RNA was extracted from sorted CD4^+^CD25^hi^CD127^low^ using the RNeasy Micro-kit (Qiagen, Venlo, The Netherlands). Concentration and purity of extracted RNA was determined using the NanoDrop spectrophotometer. RNA samples (1 µg or less) were reverse transcribed by preincubating with 5 µM oligo(dT)_16_ (Life Technologies; 10 min, 70°C), then incubation with 5x First Strand buffer, 0.1 M DTT, 10 mM dNTP and 100 U Superscript II Reverse Transcriptase (Invitrogen)(80 min, 42°C). RT-PCR reactions were as previously reported [22], [24] using StepOnePlus Real-Time PCR System and SYBR Green or Taqman systems (Applied Biosystems, Foster City, CA). Primer and probe sets were reported earlier [22], [24] and used at 400 nM and 200 nM respectively. RPL27 and B2M were used as normalization genes. The ΔΔCt method was used for data analysis.

### *In vitro* suppression assay

Stored autologous PBMCs were labeled with 1 µM CFSE (Biochimika), used as responders and stimulated with soluble anti-CD3 and anti-CD28 (1 µg/mL). Unsorted CD4^+^ and sort-purified CD4^+^CD25^high^CD127^low^ T cells were added at indicated ratios. After 72 h, cells were stained anti-CD4-PacOrange, anti-CD8-PECy7, anti-CD44-eFluor450 (eBioscience), Fixable Viability Dye eFluor780 (eBioscience). Responder proliferation was determined using CFSE dilution. The percentage of suppression was calculated as: 100×(1– [(% CFSE^dim^) in the presence of Tregs] divided by [(% CFSE^dim^) in the absence of Tregs], where CFSE^dim^ reflects cells that underwent>1 division.

### Cytokine measurement

For the quantitative detection of cytokines in the supernatant, the Human Th1/Th2/Th9/Th17/Th22 13plex Kit FlowCytomix (eBioscience) was used according to manufacturer's recommendations. Samples were measured using the Gallios flow cytometer (Beckman Coulter) and analyzed with the FlowCytomixPro Software (eBioscience).

### Statistics

The normality of all datasets was tested using D'Agostino-Pearson omnibus normality test. Where data did not significantly deviate from the normal distribution, either an independent or paired Student t test was used to test for significance as indicated. Where data were found to significantly deviate from the normal distribution, statistical significance was determined using Mann-Whitney test (unpaired data). All statistical analyses were performed using Prism (GraphPad Software, La Jolla, CA). Significance was defined as ns: not significant; *P<0.05; **P<0.01; ***P<0.001.

## Results

### Similar vitamin D status and Treg frequency in donor cohorts

Lower concentrations of serum 25(OH)D_3_, considered to be the best estimate of body stores of vitamin D [27], have been detected in children or young adults with newly-diagnosed type 1 diabetes in comparison to control subjects [17]. In this study, we found comparable serum concentrations of 25(OH)D_3_ in control donors and patients (Fig. S1 *B*). Stratification of serum 25(OH)D_3_ concentrations per season revealed an expected seasonal pattern with the highest concentrations in the summer months and lowest concentrations in the spring/winter, in line with the amount of available ultraviolet (UV) B irradiation in the preceding months (Fig. S1 *B*). Of note, the same blood samples were used to determine serum 25(OH)D_3_ concentrations as well as for immune analysis (see below).

### Exposure to 1,25(OH)_2_D_3_ or TX527 reduces production of helper T cell cytokines

We have previously shown that TX527, a low-calcemic analog of 1,25(OH)_2_D_3_, modulates the cytokine expression profiles of human T cells [22]. To determine how 1,25(OH)_2_D_3_ and TX527 affect the differentiation of T cells from type 1 diabetes patients, we measured the cytokine production by CD3^+^ T cells after culture in the presence of 1,25(OH)_2_D_3_ and TX527. For this purpose, human CD3^+^ T cells were isolated from PBMCs of control subjects and patients with type 1 diabetes, activated using anti-CD3 and anti-CD28 and cultured as described in the Research Design and Methods section. Cells were treated with vehicle (ethanol), 10^−8^ M 1,25(OH)_2_D_3_ or 10^−8^ M TX527 every other day. On 8 day, cytokine concentrations were determined in the culture supernatant using a multiplex detection system.

**Figure 1:**
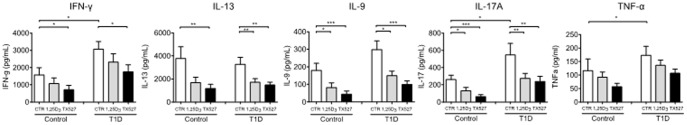
1,25(OH)_2_D_3_ and TX527 reduce T helper cytokines in human T cell cultures. Human peripheral blood CD3^+^ T cells, isolated from control subjects (n = 19) and type 1 diabetes patients (n = 20), were activated using anti-CD3/CD28 and treated with vehicle (CTR), 10^−8^ M 1,25(OH)_2_D_3_ (1,25D_3_) or 10^−8^ M TX527. Concentrations of indicated cytokines were determined in the supernatant of day 8 cultures. Results are shown as bar graphs of mean ± SEM, data are grouped per treatment and donor type. Significance was calculated using a two-tailed Mann-Whitney test. * *P*<0.05; ** *P*<0.01; *** *P*<0.001. All other comparisons were not statistically significant.

We found a higher concentration of IFN-γ, TNF-α, and IL-17A in vehicle-treated T cell cultures from donors with type 1 diabetes than in those from control donors, suggesting more cytotoxic T cells, Th1 and Th17 cells, in line with the inflammation associated with type 1 diabetes (Fig. 1). Exposure of T cells to either 1,25(OH)_2_D_3_ or TX527 reduced the production of Th1 cytokines, as demonstrated by the decrease in produced IFN-γ and TNF-α (Fig. 1). This was evident in cultures from both type 1 diabetes and control donors. Both 1,25(OH)_2_D_3_ and TX527 also reduced the Th2 cytokines IL-13 (Fig. 1) and IL-4 and IL-5 (data not shown) in cultures from control and type 1 diabetes donor T cells, albeit not always statistically significant. Moreover, production of Th9 (IL-9) and Th17 (IL-17A) cytokines by T cells from control subjects and type 1 diabetes donors were reduced after exposure to 1,25(OH)_2_D_3_ or TX527 (Fig. 1). IL-22 and IL-12 were hardly detected using this approach (data not shown). Taken together, these data show that both the natural bioactive vitamin D compound 1,25(OH)_2_D_3_ and the low-calcemic analog TX527 reduce the cytokine production irrespective of whether the T cells were isolated from type 1 diabetes patients or control donors.

### 1,25(OH)_2_D_3_ and TX527 induce receptors responsible for homing to skin and inflammation

**Figure 2:**
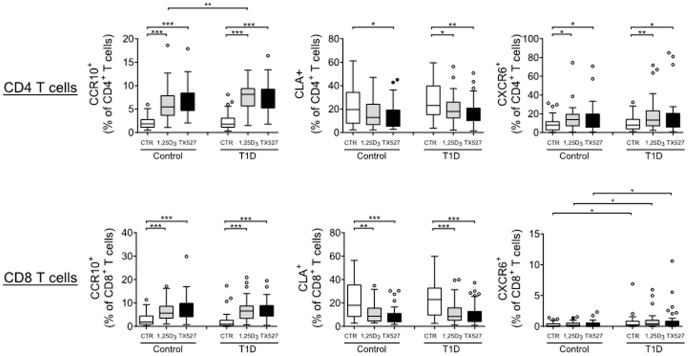
Induction of CCR10 and CXCR6 and reduction of CLA on activated T cells. Expression of CCR10, CLA or CXCR6 by CD4^+^ (**A**) and CD8^+^ T cells (**B**) after activation of T cells from control subjects (Control, n = 33) or type 1 diabetes patients (T1D, n = 45) in the presence of vehicle (CTR), 10^−8^ M 1,25(OH)_2_D_3_ (1,25D_3_) or 10^−8^ M TX527. On day 8, cells were harvested and stained for flow cytometry. Shown are box and Tukey whisker plot summarizing the frequencies of positive cells in the CD4 or CD8 gate respectively, data are grouped per donor type and treatment, cross-bar indicates median. Significance was calculated using a two-tailed Mann-Whitney test. * *P*<0.05; ** *P*<0.01; *** *P*<0.001. All other comparisons were not statistically significant.

To gain insight into the role of 1,25(OH)_2_D_3_ and TX527 as potential modulators of T cell movement, we analyzed the surface expression of the skin-homing receptors C-C chemokine receptor type 10 (CCR10) and cutaneous leukocyte-associated antigen (CLA), and the inflammation-associated homing receptor C-X-C chemokine receptor type 6 (CXCR6). We and others had previously published that 1,25(OH)_2_D_3_ or TX527 increases the expression of CCR10 in T cells from control subjects [22], [28]. On day 8, both 1,25(OH)_2_D_3_ and TX527 had induced CCR10 on CD4^+^ and CD8^+^ T cells, not only from control donors, but also from patients with type 1 diabetes (Fig. 2). On the other hand, exposure to 1,25(OH)_2_D_3_ or TX527 decreased the expression of CLA on CD4^+^ and CD8^+^ T cells from both control subjects and type 1 diabetes patients. In addition, 1,25(OH)_2_D_3_ or TX527 triggered elevated expression of CXCR6, a chemokine receptor guiding T cells to sites of inflammation, on CD4^+^ T cells, but did not influence CXCR6 expression on CD8^+^ T cells. Overall, these data suggest that 1,25(OH)_2_D_3_ or TX527 alters the migratory behavior of human T cells towards chemokine ligands by modulating their homing receptor expression profile. This may assist proper homing of conditioned T cells upon adoptive transfer *in vivo*.

### Exposure to 1,25(OH)_2_D_3_ or TX527 promotes a Treg phenotype

We found a similar CD4-to-CD8 ratio and a similar frequency of CD4^+^CD25^high^CD127^low^ T cells in PBMCs freshly isolated from control subjects or from patients with type 1 diabetes (Fig. S2). It is important with regards to therapeutic application of *ex vivo*-expanded autologous Treg to investigate whether triggering the VDR pathway also increases Tregs in T cells from patients with type 1 diabetes, similar to what we had shown in human CD3^+^ T cells from control subjects [22].

**Figure 3:**
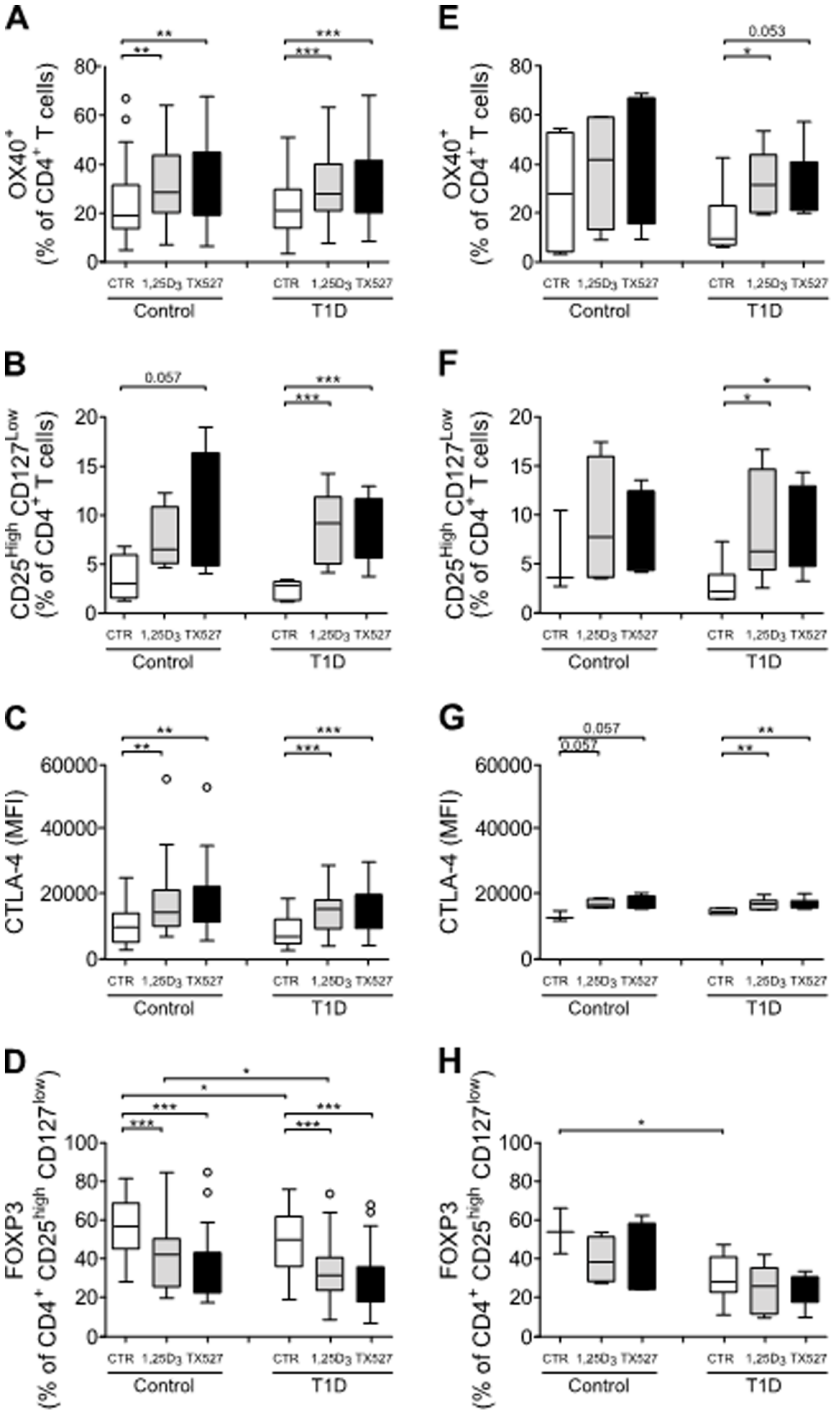
1,25(OH)_2_D_3_ and TX527 trigger a stable Treg phenotype in T cells from human type 1 diabetic patients. T cells from control subjects (Control) or type 1 diabetes patients (T1D) were cultured in the presence of vehicle (CTR; white boxes), 10^−8^ M 1,25(OH)_2_D_3_ (1,25D_3_, grey boxes) or 10^−8^ M TX527 (black boxes). On day 6, the T cell cultures were exposed to normal T cell medium (left panel: **A-D**) or a cytokine cocktail (right panel: **E-H**) as described in the methods section. T cells were harvested 48 h later and stained for flow cytometry. Box and Tukey wisker plot summarizes the frequencies of positive cells in the CD4+ T cell gate. **A**: Surface expression of OX-40 (CD134) by activated CD4^+^ T cells of control donors (Control, n = 43) or type 1 diabetes patients (T1D, n = 58). **B**: Frequency of CD25^high^CD127^low^ cells in the CD4^+^ T cell gate from control subjects (Control, n = 4) and type 1 diabetes patients (T1D, n = 7). CTLA-4 (**C**) and FOXP3 (**D**) expression in CD4^+^CD25^high^CD127^low^ T cells of control donors (Control, n = 28) and type 1 diabetes patients (T1D, n = 45). **E**: Frequency of OX-40 expression on CD4^+^ T cells from control subjects (Control, n = ) or type 1 diabetes patients (T1D, n = 7) after additional stimulation with a cytokine cocktail. **F**: Frequency of CD25^high^CD127^low^ cells in CD4^+^ T cells. CTLA-4 (**G**) and FOXP3 (**H**) expression in the CD4^+^ CD25^high^CD127^low^ T cell gate. Data are grouped per donor type and treatment, cross-bars indicate median ± SEM. Significance was calculated using a two-tailed Mann-Whitney test. * *P*<0.05; ** *P*<0.01; *** *P*<0.001. All other comparisons were not significantly different.

A first indication that VDR agonists such as 1,25(OH)_2_D_3_ also induce a Treg phenotype in T cells from type 1 diabetes patients came from our data on CD134 (OX-40), a secondary co-stimulatory molecule that is constitutively expressed in naturally occurring CD4^+^ Tregs (nTregs) in mice and upregulated on the surface of human nTregs upon TCR cross-linking [29]. We found increased frequencies of OX-40-expressing CD4^+^ T cells after exposure of T cells from patients with type 1 diabetes to 1,25(OH)_2_D_3_ or TX527 (Fig. 3*A*), suggesting increased presence of Tregs. Indeed, assessment of surface expression of CD25 and CD127 on CD4^+^ T cells by flow cytometry showed that 1,25(OH)_2_D_3_ and TX527 increased the frequency of CD4^+^CD25^high^CD127^low^ T cells in cultures of human T cells from patients with type 1 diabetes (Fig. 3*B*). In addition, 1,25(OH)_2_D_3_ and TX527 were equally powerful at inducing this effect. We then analyzed the expression of molecules involved in Treg development and function. Within the CD4^+^CD25^high^CD127^low^ T cell population, 1,25(OH)_2_D_3_ or TX527 increased the expression of the Treg-associated molecule cytotoxic T lymphocyte antigen 4 (CTLA-4, CD152) in T cells of control subjects and patients with type 1 diabetes (Fig. 3*C*). Somewhat surprisingly, FOXP3, a transcription factor that plays a key role in Treg development and function [30], was reduced after exposure to 1,25(OH)_2_D_3_ or TX527 in the CD4^+^CD25^high^CD127^low^ T cell population, both in frequency of FOXP3^+^ cells (Fig. 3*D*) and the FOXP3 expression levels (data not shown). It is important to emphasize that these ‘vitamin D effects’ were observed in T cells from each individual donor, a phenomenon that remains underrepresented in the box plots shown. Taken together, these observations led to the conclusion that *in vitro*, VDR agonists can promote expansion of CD4^+^CD25^high^CD127^low^ Tregs in control subjects and patients with type 1 diabetes. One of the major concerns for Treg therapy is that they may change their phenotype upon encountering an inflammatory environment. To address this issue, end-stage 1,25(OH)_2_D_3_- or TX527-treated cultures were exposed to a cocktail of proinflammatory cytokines known to promote effector T cell reactivity [25], [26]. Strikingly we showed a similar upward trend in the frequency of OX-40-expressing 1,25(OH)_2_D_3_- or TX527-treated CD4^+^ T cells from patients with type 1 diabetes (Fig. 3*E*) even after additional stimulation with such a robust cytokine cocktail. Supporting this finding, elevated numbers of CD4^+^CD25^high^CD127^low^ T cells in cultures of 1,25(OH)_2_D_3_- or TX527-treated T cells from patients with type 1 diabetes were detected, thereby confirming the increased presence of Tregs after cytokine exposure (Fig. 3*F*). Finally, also markers involved in Treg function such as CTLA-4 (Fig. 3*G*) and FOXP3 (Fig. 3*H*) were similarly regulated by 1,25(OH)_2_D_3_ or TX527 in T cells from patients with type 1 diabetes after additional proinflammatory cytokine stimulation. Combined, these data support a stable regulation of the promoted Treg phenotype by 1,25(OH)_2_D_3_ as well as TX527.

### 1,25(OH)_2_D_3_ and TX527 decrease IFN-γ, IL-4 and IL-17 and increase IL-10 in sorted Tregs

To examine whether administration of 1,25(OH)_2_D_3_ or TX527 directly influenced T cells, we analyzed the expression of VDR and vitamin D-related genes directly in Treg populations. For this purpose, CD3^+^ T cells were cultured as described above and CD4^+^CD25^high^CD127^low^ T cells were sorted at the end of the culture. We found that treatment with 1,25(OH)_2_D_3_ or TX527 strongly induced the direct vitamin D-target gene 24-hydroxylase (24-OHase, known as CYP24A1) and slightly increased expression of VDR (data not shown), demonstrating a direct regulation by ligand (1,25(OH)_2_D_3_ or TX527)-bound VDR in the CD4^+^CD25^high^CD127^low^ T cells.

**Figure 4:**
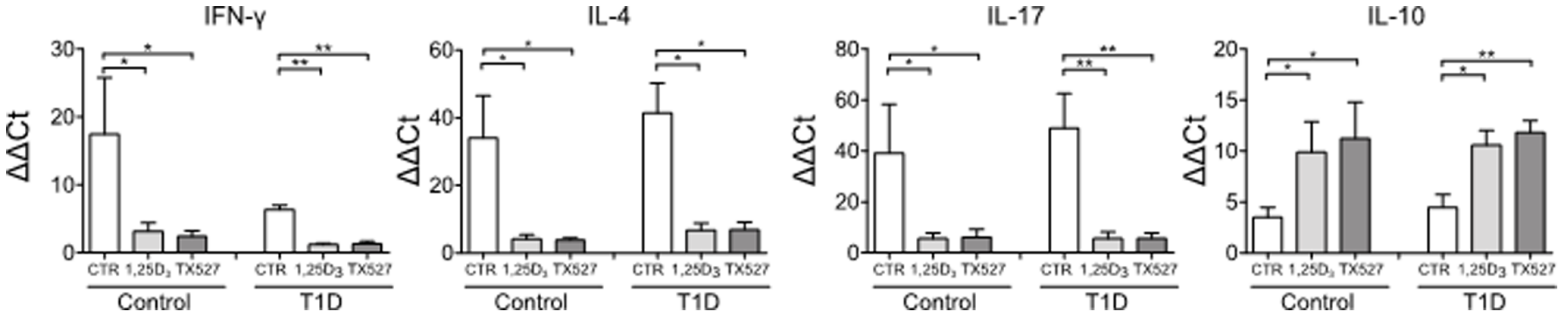
1,25(OH)_2_D_3_ and TX527 reduce IFN-γ, IL-4, and IL-17 but increase IL-10 in expanded human CD4^+^CD25^high^CD127^low^ T cells. Peripheral blood CD3^+^ T cells from control donors (n = 5) or type 1 diabetes patients (n = ) were cultured for 8 days in the presence of 10^-8^ M 1,25(OH)_2_D_3_ (1,25D_3_) or TX527 or corresponding concentration of vehicle (CTR). CD4^+^CD25^high^CD127^low^ T cells were sort-purified and mRNA expression of IFN-γ, IL-4, IL-17, and IL-10 was quantified by real-time RT-PCR using B2M and RPL27 as normalization genes. Bar graphs represent the mean ± SEM. Significance was tested using a two-tailed Mann-Whitney test. *P<0.05; **P<0.01. All other comparisons were not statistically significant.

In addition, we analyzed the effects of 1,25(OH)_2_D_3_ or TX527 on the T cell cytokine profile of human CD4^+^CD25^high^CD127^low^ T cells. In line with the cytokine production in the supernatant of bulk T cell cultures (Fig. 1), exposure to 1,25(OH)_2_D_3_ and TX527 almost completely abrogated the Th1, Th2 and Th17 cytokine function of CD4^+^CD25^high^CD127^low^ T cells, as demonstrated by the near basal levels of IFN- γ, IL-4 and IL-17A mRNA, respectively (Fig. 4). Importantly, 1,25(OH)_2_D_3_ and TX527 did not inhibit T cell cytokine production across the board, because a specific upregulation of the immunoregulatory cytokine IL-10 was observed within the CD4^+^CD25^high^CD127^low^ T cell subset (Fig. 4), whereas expression of IL-2 and TGF- β remained unchanged (data not shown). This suggested that the Tr1 cytokine activity is increased in the expanded Treg populations, both from control donors and from patients with type 1 diabetes. Taken together, 1,25(OH)_2_D_3_ and TX527 affected transcription of previously described immune-related vitamin D targets, indicating immunomodulatory actions on CD4^+^CD25^high^CD127^low^ T cells independent of antigen presenting cells.

### Unsorted CD4^+^ and sorted CD4^+^CD25^high^CD127^low^ T cells retain suppressive capacity after culture with 1,25(OH)_2_D_3_ or TX527

**Figure 5:**
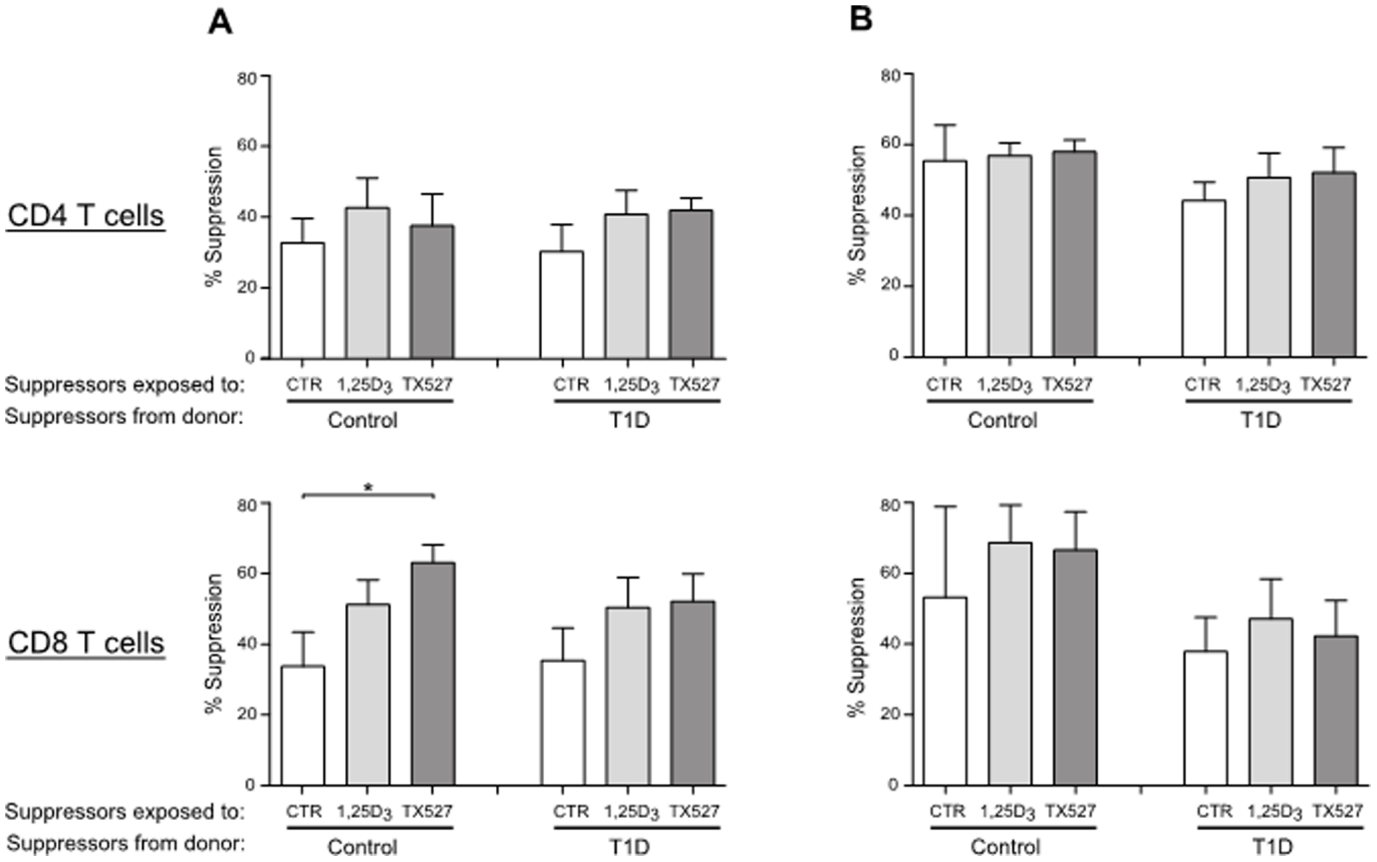
1,25(OH)_2_D_3_- or TX527-exposed human T cells from control donors and type 1 diabetes patients can suppress autologous CD4 and CD8 T cell responses. **A, B**: CFSE-labeled responder cells from control (Control, n = 5–7) and type 1 diabetes (T1D, n = 7–10) donors were stimulated for 4 days with anti-CD3/CD28 mAbs and co-cultured with autologous unsorted CD4^+^ (**A**) or sorted CD4^+^CD25^high^CD127^low^ (**B**) T cell populations (day 8) from control-, 1,25(OH)_2_D_3_- or TX527-treated cultures, as indicated. Shown are bar graphs summarizing the percentage suppression of proliferation (see Methods section) of CD4^+^ (top) and CD8^+^ (bottom) T cells without or with Tregs at a 2∶1 (in case of unsorted CD4^+^ T cells, **A**) or 1∶1 (in case of sorted CD4^+^CD25^high^CD127^low^ T cells, **B**) Treg:Tresponder ratio. Significance was tested using a two-tailed Mann-Whitney test, all not significantly different. * *P*<0.05. All other comparisons were not statistically significant.

We next tested whether 1,25(OH)_2_D_3_- or TX527-induced Tregs exhibit functional capacity to suppress autologous T cell responses. When adding the unsorted CD4^+^ T cells from vehicle-, 1,25(OH)_2_D_3_-, or TX527-treated cultures to CFSE-labeled autologous PBMC responder cells in a standard suppression assay, a higher suppression of both CD4^+^ and CD8^+^ T cell populations was seen than with vehicle-treated cells, with similar effects in immune cells from control subjects or patients with type 1 diabetes (Fig. 5A). However, when sorted CD4^+^CD25^high^CD127^low^ T cells from vehicle-, 1,25(OH)_2_D_3_-, or TX527-treated cultures were added to the *in vitro* suppressor assay, this difference was diminished (Figure 5B), suggesting that 1,25(OH)_2_D_3_- or TX527-induced Tregs were not more potent on a cell-by-cell basis, but more frequent in numbers. This is in line with our observation that more CD4^+^CD25^high^CD127^low^ T cells were recovered from 1,25(OH)_2_D_3_- or TX527-treated cultures than from untreated cultures (data not shown). However, the effect of 1,25(OH)_2_D_3_ or TX527 on Treg induction was identical for control subjects and patients with type 1 diabetes.

## Discussion

In recent years, interest in the tolerance-inducing potential of vitamin D and its analogs to modulate immune cells has grown, but one of the major obstacles for the use of active vitamin D is the need for supra-physiological doses to modulate immune responses, because of side-effects such as hypercalcemia, hypercalciuria and kidney stones. The use of structural analogs of 1,25(OH)_2_D_3_ with reduced calcemic effects but similar immunoregulatory activity might overcome this issue, but no clinical (safety) data are available. Therefore, we opted to *ex vivo* manipulate T cells with 1,25(OH)_2_D_3_ for subsequent use in autologous adoptive immunotherapy. We recently demonstrated that *ex vivo* treatment of human T cells from healthy individuals with TX527, a vitamin D analog with reduced calcemic side effects, triggers the emergence of a CD4^+^CD25^high^CD127^low^ T cell population [22]. To further explore the clinical applicability of 1,25(OH)_2_D_3_ and TX527 in the context of autologous adoptive T cell therapy, we show here that 1,25(OH)_2_D_3_ or TX527 can also imprint T cells from type 1 diabetes patients with a stable regulatory profile (even in the presence of a severe systemic inflammatory response) and a suppressive capacity on autologous T cells. These results are particularly relevant to the use of Tregs as an adoptive cell therapy.

Many immunotherapies have failed to dampen the autoimmune process in type 1 diabetes without simultaneously affecting normal immunity. The emergence of Tregs as central mediators of peripheral tolerance in the immune system has led to an important area of clinical investigation to target these cells for the treatment of autoimmune diseases such as type 1 diabetes. Deficits in the number and/or suppressive activity of Tregs, which have been implicated in sustaining immune homeostasis, have been postulated as underlying mechanisms by which individuals develop type 1 diabetes. However, to date, there is controversy about whether Treg frequency and/or function are impaired at type 1 diabetes onset [6]–[10], [31], [32]. In line with previous findings [7], [8], we have confirmed normal CD4^+^/CD8^+^ T cell ratios and the Treg frequencies in our type 1 diabetes patient cohort. Increasing the frequency and/or function of Tregs has been proposed as a potential therapy in type 1 diabetes and can be achieved by either targeting these cells directly *in vivo* or by expanding them *ex vivo* prior to re-introduction in the patient. Currently, a phase 1 clinical trial of CD4^+^CD25^+^CD127^low/-^ polyclonal Tregs expanded using anti-CD3 and anti-CD28 coated microbeads and IL-2 is ongoing (NCT01210664). While we did not directly compare the Treg expansion protocol used with our approach, we believe that addition of active vitamin D (analog) might benefit *ex vivo* expansion protocols because it imprints the cells with a migratory signature towards sites of inflammation [22], reduces pro-inflammatory cytokine production and induces anti-inflammatory IL10. More specifically, our results showed a similar 1,25(OH)_2_D_3_- and TX527-mediated modulation of CCR10 and CLA expression, in line with a previous report showing that CCR10 expression increased on human CD4^+^ and CD8^+^ T cells upon activation in the presence of 1,25(OH)_2_D_3_ [22], [24], [28], [33]. It is also important in the context of type 1 diabetes, which is associated with IFN-γ producing Th1 cells, IL-17A producing Th17 cells and an increase in IL-9^+^IL-17^+^ cells, that exposure of T cells from type 1 diabetes donors to 1,25(OH)_2_D_3_ and TX527 reduced production of all tested helper T cell cytokines. This inhibitory effect on Th1 (IFN-γ) and Th17 (IL-17) production is also consistent with a variety of other studies regarding the direct effect of VDR agonist on mouse and human T cells [21], [22], [24], [34]–[36]. For example, stimulation of CD4^+^CD25^-^ human T cells with anti-CD3/CD28 beads in the presence of 1,25(OH)_2_D_3_ inhibited the production of inflammatory cytokines, such as IFN-γ, IL-17 and IL-21 [21]. Exposure of human T cells to 1,25(OH)_2_D_3_ and TX527 increased IL-10 mRNA in sorted CD4^+^CD25^high^CD127^low^ T cells, in line published data on increased IL-10 production after *in vitro* exposure of mouse and human T cells to VDR agonists [21], [22], [37].

Intriguingly, the vitamin D-induced Tregs exhibited the capacity to suppress proliferation of autologous responder T cells. Because it is clinically only relevant to know whether Tregs from T1D patients can suppress autologous effector T cell responses, we did not evaluate whether Tregs generated from T1D patients are relatively more or less potent than those from control donors using cross-over experiments. Instead we showed elevated levels of regulatory mediators such as IL-10, TGF-β and CTLA-4 but decreased frequencies of FOXP3^+^ cells, even in high inflammatory settings. Although FOXP3 has been considered as a lineage-specifying master regulator of Treg functions [30], several indications that FOXP3 expression per se might not be sufficient to stably maintain Treg suppressive function or reliably delineate functional Tregs. For example, activated human effector T cells can transiently express FOXP3 at a low level without acquiring suppressive activity [38]. Also, human peripheral blood CD4^+^ T cells contain a FOXP3^+^ T cell subpopulation that does not exhibit suppressive activity and even produces proinflammatory cytokines upon activation [39]. Finally, FOXP3 has a transient expression pattern in the presence of 1,25(OH)_2_D_3_ [21], suggesting that vitamin D-induced Tregs do not depend on FOXP3. In a recent study, treatment of human CD4^+^ T cells with 1,25(OH)_2_D_3_ in combination with IL-2 promoted the development of IL-10 producing CTLA-4^+^FOXP3^+^ T cells with suppressive capacity [21], and we and others showed increased levels of the regulatory cytokine IL-10 [22], [40]. *In vivo*, IL-10 was identified as an essential component for vitamin D-mediated inhibition of experimental autoimmune encephalomyelitis [41] and *in vitro*, vitamin D-induced IL-10-producing Tregs were able to prevent the induction of experimental autoimmune encephalomyelitis in an IL-10 dependent way [40]. Future research will assess the role of IL10 in the suppressive function of our cultured cells, their stability using FOXP3 TSDR demethylation analysis [42] and whether gene polymorphisms of vitamin D genes or type 1 diabetes susceptibility genes influence the potential of vitamin D to imprint Treg phenotype and/or function.

In conclusion, our data show that *ex vivo* exposure to VDR agonists – 1,25(OH)_2_D_3_ and the low-calcemic analog TX527 – can increase the numbers of functional IL-10 producing CD4^+^CD25^high^CD127^low^ T cells with a stable phenotype and imprint T cells with a unique homing signature and reduced production of effector cytokines. The possibility to induce functional Tregs from T cells isolated from type 1 diabetes patients warrants additional validation of vitamin D-induced Tregs for autologous adoptive immunotherapy in type 1 diabetes.

## Supporting Information

Figure S1 **Demographic information of the study participants.** **A**: Summary of the gender, age (in years as average ± SD), number (n) of control subjects and patients with type 1 diabetes participating in the study. **B**: Vitamin D status measured as 25(OH)D_3_ concentration in serum using 25(OH)D_3_ DiaSorin RIA kit. Not all participants were included in every single assessment described in this study.
(TIF)

Figure S2 **Similar CD4/CD8 ratio and frequency of Tregs in PBMC isolated freshly from control subject and patients with type 1 diabetes (T1D).** **A**: Flow cytometry data (top) of percentage CD4^+^ and CD8^+^ T cells gated on viable CD3^+^ T cells in isolated PBMCs and cumulative data per donor group (bottom) of the ratio of CD4^+^ to CD8^+^ T cells. **B and C**: Flow cytometry plots (top) of CD127 versus CD25, gated on viable CD4^+^ T cells (**B**) or CD8^+^ T cells (**C**). Gates were drawn to determine the fractions of CD25^high^CD127^low^ cells that are used in the cumulative data graph (bottom, control: n = 41, T1D: n = 49). Plots shown are from one representative control individual. In the cumulative graphs, each symbol represents an individual donor. Horizontal lines indicate the mean ± SEM. Data are pooled from multiple measurements at different time points. Statistical analysis of values between controls and T1D patients was performed using a two-tailed Mann-Whitney test. ns: not significant.
(TIF)
