# Supplementary figures and images for: 1,25-Dihydroxyvitamin D3 and Its Analog TX527 Promote a Stable Regulatory T Cell Phenotype in T Cells from Type 1 Diabetes Patients
Source: PLoS One. 2014 Oct 3;9(10):e109194. doi: 10.1371/journal.pone.0109194 (PMC4184870; doi:10.1371/journal.pone.0109194)

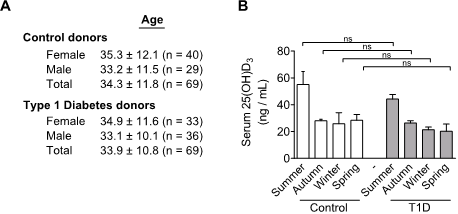

Supplement: Figure S1 — Demographic information of the study participants. A: Summary of the gender, age (in years as average ± SD), number (n) of control subjects and patients with type 1 diabetes participating in the study. B: Vitamin D status measured as 25(OH)D3 concentration in serum using 25(OH)D3 DiaSorin RIA kit. Not all participants were included in every single assessment described in this study. (TIF) [file pone.0109194.s001.tif]

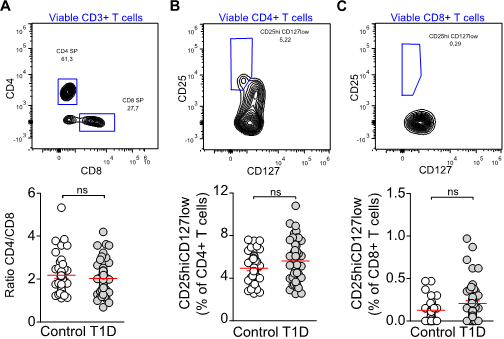

Supplement: Figure S2 — Similar CD4/CD8 ratio and frequency of Tregs in PBMC isolated freshly from control subject and patients with type 1 diabetes (T1D). A: Flow cytometry data (top) of percentage CD4+ and CD8+ T cells gated on viable CD3+ T cells in isolated PBMCs and cumulative data per donor group (bottom) of the ratio of CD4+ to CD8+ T cells. B and C: Flow cytometry plots (top) of CD127 versus CD25, gated on viable CD4+ T cells (B) or CD8+ T cells (C). Gates were drawn to determine the fractions of CD25highCD127low cells that are used in the cumulative data graph (bottom, control: n = 41, T1D: n = 49). Plots shown are from one representative control individual. In the cumulative graphs, each symbol represents an individual donor. Horizontal lines indicate the mean ± SEM. Data are pooled from multiple measurements at different time points. Statistical analysis of values between controls and T1D patients was performed using a two-tailed Mann-Whitney test. ns: not significant. (TIF) [file pone.0109194.s002.tif]
